# Genome-Wide Identification and Characterization of *CIPK* Family and Analysis Responses to Various Stresses in Apple (*Malus domestica*)

**DOI:** 10.3390/ijms19072131

**Published:** 2018-07-22

**Authors:** Lili Niu, Biying Dong, Zhihua Song, Dong Meng, Yujie Fu

**Affiliations:** 1 Key Laboratory of Forest Plant Ecology, Ministry of Education, Northeast Forestry University, Harbin 150040, China; niulili0613@126.com; 2 The College of Forestry, Beijing Forestry University, Beijing 100083, China; dongbiying1029@163.com (B.D.); szhxf0801@163.com (Z.S.); 3 Beijing Advanced Innovation Center for Tree Breeding by Molecular Design, Beijing Forestry University, Beijing 100083, China

**Keywords:** *Malus domestica*, *CIPK* genes, phylogeny, expression pattern, stress

## Abstract

In the *CIPK* family, the CBL-interacting protein kinases have shown crucial roles in hormone signaling transduction, and response to abiotic stress in plant developmental processes. The *CIPK* family is characterized by conserved NAF/FISL (Asn-Ala-Phe) and PPI (protein-phosphatase interaction) domains in the C-terminus. However, little data has been reported about the *CIPK* family in apple. A total of 34 *MdCIPK* genes were identified from the apple genome in this study and were later divided into two groups according to the CIPK domains, characterized by gene structure and chromosomal distribution, and then mapped onto 17 chromosomes. All *MdCIPK* genes were expressed in the four apple tissues (leaf, root, flower, and fruit). In addition, the *MdCIPK* gene expression profile showed that five members among them revealed enhanced expression during the pollen tube growth stages. The *MdCIPK*4 was the most expressive during the entire fruit development stages. Under stress conditions 21 *MdCIPK* genes transcript levels were up-regulated in response to fungal and salt treatments. This suggested the possible features of these genes’ response to stresses in apples. Our findings provide a new insight about the roles of *CIPK* genes in apples, which could contribute to the cloning and functional analysis of *CIPK* genes in the future.

## 1. Introduction

Calcium as the second messenger plays a crucial role in regulating plant stress signaling pathways during plant development [1]. The intracellular calcium levels are regulated when plants respond to exogenous stimuli, biotic stress, abiotic stress, and perform physiological activities [2,3]. Stress-induced Ca^2+^ signals are decoded by many Ca^2+^-sensors including calmodulins, calcineurin B-like proteins (CBLs), and calmodulin-binding proteins [4,5]. CBLs specifically target the *CIPK* family, which contains the CBL-interacting protein kinases, and regulate their downstream genes resulting in a series of physiological and biochemical changes [6]. CIPK protein is a plant-specific Ser/Thr protein kinase and belongs to the plant SnRK3 protein kinase subfamily. The major structural domains in CIPK consist of N-terminal kinase (PKC) and C-terminal regulatory domain. The N-terminal domain is similar in protein structure to SNF1 kinase and AMO-dependent protein kinase. The C-terminal regulatory domain is unique and very conserved to the *CIPK* family [7,8].

*CIPK* genes were specifically expressed in plants and participated in the growth and development of plant tissues and organs. For example, the *TaCIPK* genes showed up-regulated or down-regulated expressions during seed germination in wheat [9]. Some genes were specifically and abundantly expressed in certain developmental stages of turnips. This implied that these genes may play an important role during developmental stages [10]. There have been reports that *CIPK* genes play critical roles in response to various biotic and abiotic stresses such as heat, drought, salinity, and freezing stresses [11,12,13,14]. In legume the transcript levels of *PsCIPK* were stimulated to change in response to calcium and salicylic acid stress. However, the expression of these genes had no effect on response to drought or abscisic acid stress [8].

As an important economic tree, apples (*Malus domestica*) are one of the most widely cultivated crops in the world [15]. Furthermore, the apple genome sequence has been reported [16]. More genome sequencing has been completed, and many *CIPK* genes have been identified and characterized. It has been reported that there were 26 *CIPK*s that have been identified in *Arabidopsis* [17], 43 *CIPK*s in maize [18], 34 *CIPK*s have been identified in rice [19,20], 27 *CIPK*s in poplar [21], 25 *CIPK*s in cassava [22], 28 *CIPK* family members from pear [23], 32 *CIPK*s in sweet sorghum [24], and 51 *CIPK*s in turnip (*Brassica rapa* var. *rapa*) [10]. However, only a few *CIPK* genes have been discovered in apple. *MdCIPK24-LIKE1* has been identified for increased antioxidant metabolites and improved salt tolerance in apple [25]. The *MdCIPK*6*L* gene, expression was positively induced by different stresses in apple [26].

As one of the woody perennial species, the apple tree often suffers from a variety of environmental stresses making it urgent to comprehend the function of the *CIPK* genes in stress responses in order to improve crop tolerances against adverse environments [27]. In this study, all the *CIPK* genes were identified by apple genome-wide analysis, their evolutionary relationship phylogenetically was evaluated, and their structure and chromosomal distribution of the *CIPK* genes were analyzed. In addition, based on our RNA-seq data the *MdCIPK* gene expression profiles in different apple tissue, fruit development stages, and pollen tube growth stages were determined. Furthermore, the influence of biotic and abiotic stresses on their expression levels were also analyzed. The results in this study offer a thorough understanding for *CIPK* genes in apple and provide scientific reference for genetic breeding purposes.

## 2. Results

### 2.1. Identification of CIPK Family Genes in Apple

**Figure 1:**
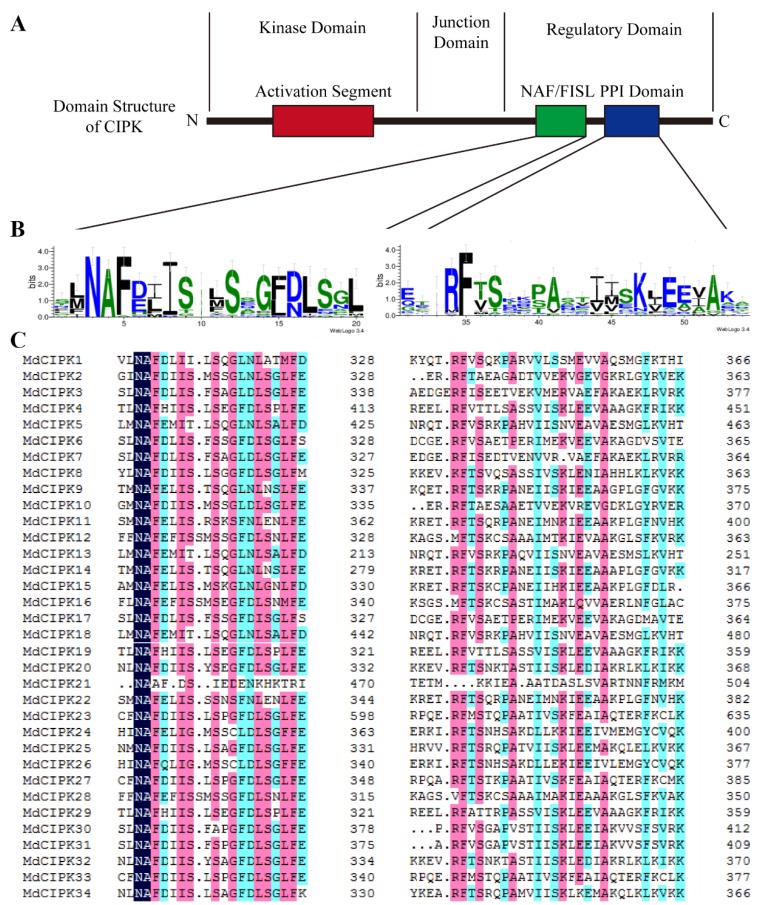
CIPK domains in the MdCIPK proteins. (**A**). The conserved domains were identified using the MEME suite. (**B**). The overall height in each stack indicates the sequence conservation at each position. Each residue letter height is proportional to the relative frequency of the corresponding residue. (**C**). The CIPK domain of each group was compared use the DNAMAN 7.0 software and conserved amino acid residues are shown in dark blue. Sequences highlighted in dark blue, red, and light blue indicate homology =100%, ≥75%, and ≥50%, respectively.

The Arabidopsis CIPK proteins were used as query sequences to identify the *CIPK* genes in the apple genome. In this study, a total of 34 typical *CIPK* genes were identified and annotated in apple (Table 1). According to the different gene coordinate orders on apple chromosomes, 34 apple *MdCIPK* genes were named from *MdCIPK*1 to 34. As shown in Table 1 the molecular weights of these 34 MdCIPKs were deduced to a range from 36.72 kDa (MdCIPK13) to 80.86 kDa (MdCIPK23). All 34 *MdCIPK* genes lengths ranged from 322 (*MdCIPK*13) amino acids (AAs) to 729 (*MdCIPK*23) AAs. In addition, the isoelectric points of these proteins were predicted to be between 6.14 (MdCIPK15) and 9.68 (MdCIPK4). Kinase, NAF, and PPI domains; which are the basic characteristics of CIPK family. Conserved domain analysis confirmed that all 34 MdCIPKs identified the kinase, NAF, and PPI domains in apple (Figure 1).

### 2.2. Phylogenetic Analysis of the MdCIPK Family Genes

**Figure 2:**
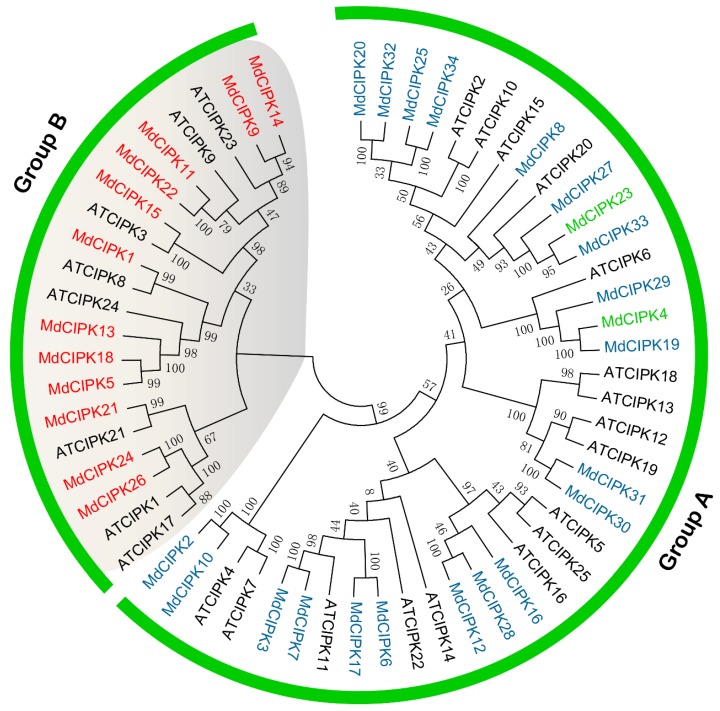
Phylogenetic relationship of apple and Arabidopsis CIPK proteins. The phylogenetic tree was constructed using the neighbor—joining method by MEGA 6.0. The bootstrap values of 1000 replicates were calculated at each node. The model was p-distance and the pattern among Lineages was Same (Homogeneous). The gaps and missing data treatment were complete deletion. The CIPK domain protein sequences, 34 from apple (MdCIPKs) and 25 from Arabidopsis (AtCIPKs, black color), were aligned by MUSCLE. The proteins were classified into 2 distinct subgroups, Group A and Group B. Red indicates *MdCIPK* genes with exon-poor in Group B, blue indicates *MdCIPK* genes with no introns in Group A, and green indicates *MdCIPK* genes containing introns in Group A.

In this study, a total 34 *CIPK* genes were identified in apple. Phylogenetic analysis was performed to investigate the evolutionary relationships within all the *CIPK* genes in apple. 25 Arabidopsis CIPK proteins and 34 apple CIPK proteins were compared using the MEGA 6.0 Program (Figure 2). The phylogenetic tree of the *MdCIPK*s was divided into two groups (A and B) of monophyletic clades. Group A contained 22 *MdCIPK* genes, and Group B contained 12 *MdCIPK* genes. The result suggested that the 34 *CIPK* genes can be classified into two groups in apple based on their sequence similarity. Similar results have been found in Arabidopsis and Populus [21].

### 2.3. Gene Structure of CIPK Genes in Apple

**Figure 3:**
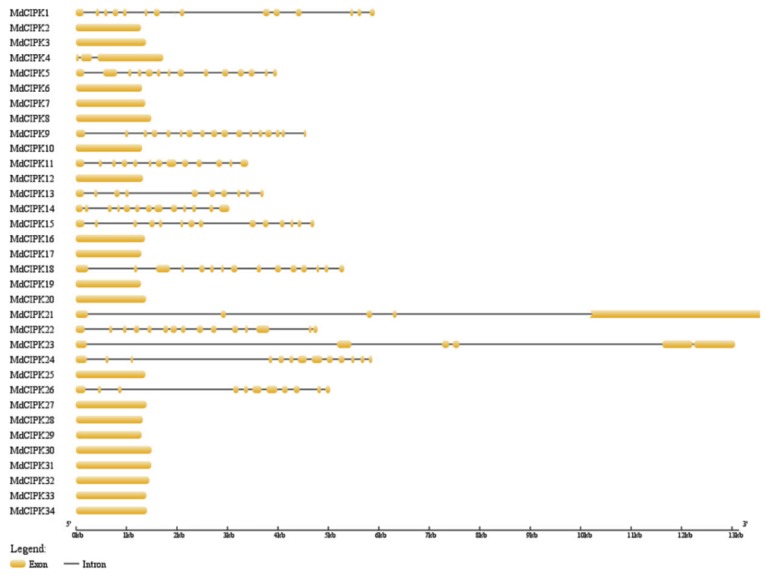
The exon-intron structure analyses of *CIPK* family in apple. Lengths of exons and introns of each *MdCIPK* gene were exhibited proportionally.

The *MdCIPK* genes in apple the exon-intron structure of *MdCIPK* genes have been analyzed to determine their structural diversity. From Figure 3, among the 34 *MdCIPK* genes there were 20 without introns, yet the remaining 14 genes contained more introns. Interestingly, the phylogenetic analysis indicated that 34 *MdCPK* genes were divided into two groups (A and B; Figure 2). A total of 20 *MdCIPK* genes were rich in exons, and clustered into group A. However, the exons-poor genes were clustered into the other group B (Figure 2). Similar gene structures appeared in the same group and were closely related to each other. It was reported that *CIPK* genes also divided into two groups in Arabidopsis, which possessed genes with no introns and exon-poor respectively [17]. Some similar results for *CIPK* genes were also observed in cassava, pear, and grapevine which indicated that the genetic structure of the *CIPK* family was conserved.

### 2.4. Chromosomal Distribution of the MdCIPK Family

**Figure 4:**
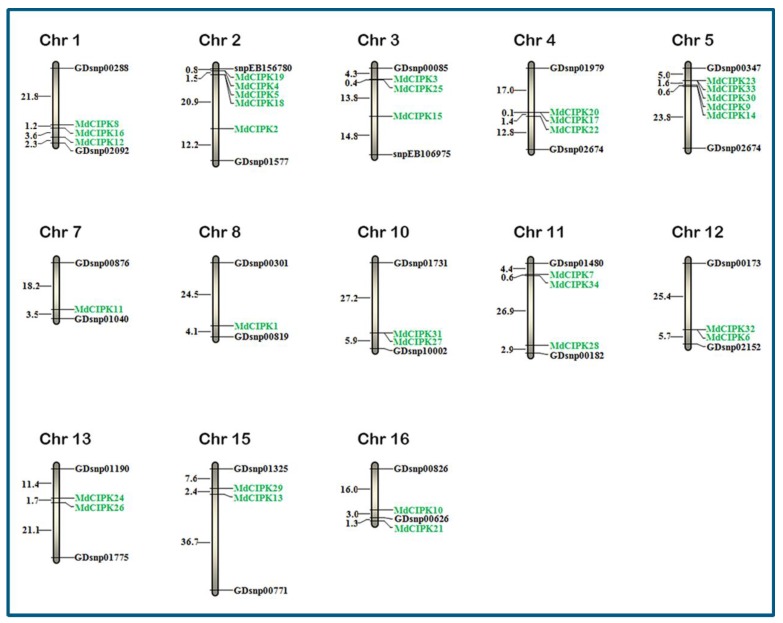
The distribution of *MdCIPK* genes in apple chromosomes (green color). There were 4 chromosomes that 6, 9, 14, and 17 chromosomes with no *MdCIPK* genes that not shown in this picture.

The *MdCIPK* genes in apple the location of *MdCIPK*s were marked on the chromosomes to investigate the genomic distribution. As shown in Figure 4, the chromosomal location analysis revealed that the 34 *MdCIPK* genes were mapped onto a total 17 apple chromosomes; however, the distribution of *MdCIPK* genes in chromosomes was uneven. Chromosome 7 and 8 contained the least number of *MdCIPK* genes with the only one *CIPK1* and *CIPK11* gene, respectively. Chromosomes 10, 12, 13, 15, and 16 have two *MdCIPK* genes, moreover chromosomes 1, 3, 4, and 11 included three *MdCIPK* genes respectively.

Chromosomes 2 and 5 both had 5 *CIPK* genes. However, no *MdCIPK* genes mapped onto the 6, 9, 14, and 17 chromosomes. The similar phenomenon for chromosomal distribution of *CIPK* genes was also observed in the grapevine; there was no *CIPK* gene found on chromosomes 14 and 17, and also no *CIPK* gene located on grape chromosomes 1, 3, 7, and 12 [28]. The results indicated that an unbalanced distribution of *MdCIPK* genes on chromosomes may be related to species evolution and genetic variation.

### 2.5. Expression Patterns of MdCIPK Genes in Different Tissues

**Figure 5:**
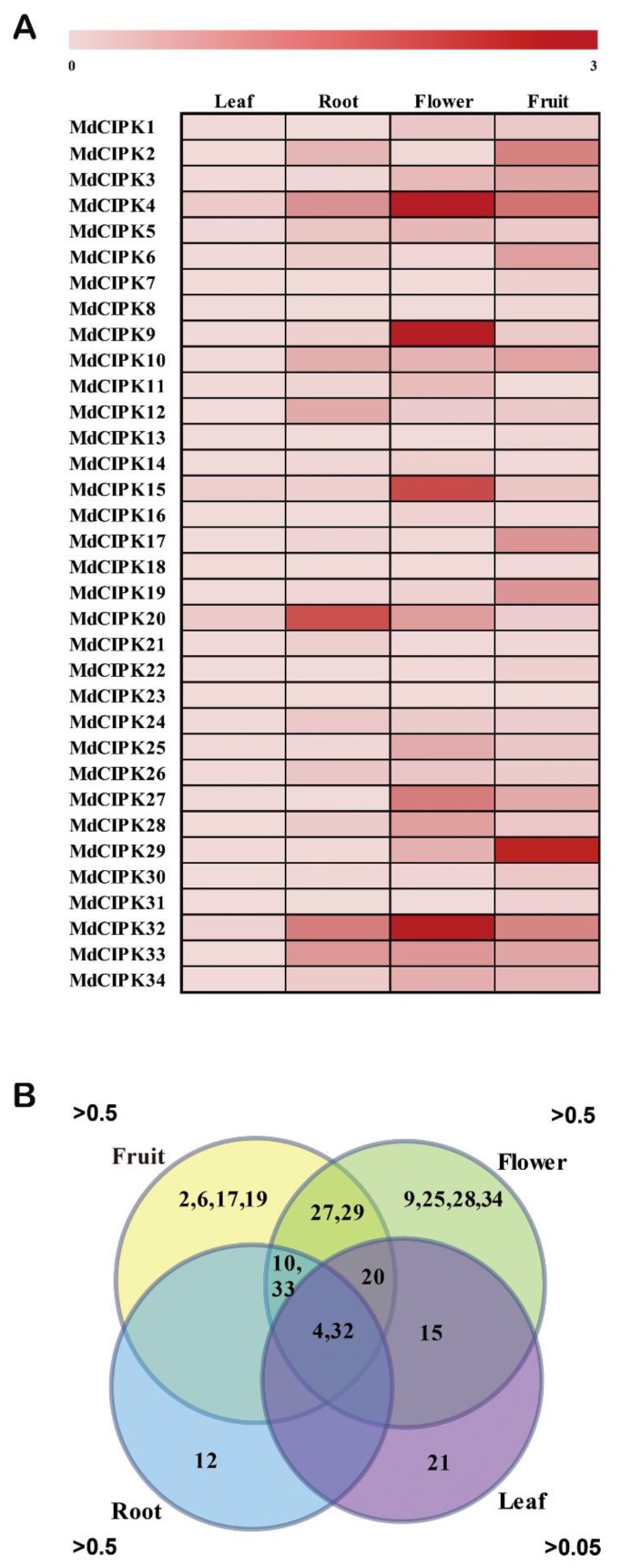
Expression patterns of apple *CIPK* genes in different tissues. Heat map of the 34 *MdCIPK* genes expression patterns in different apple tissues (**A**) and Venn diagram of differentially expressed gene sets, the numbers in each section represented *MdCIPK* genes (**B**). Three replicates were performed for each organ.

Transcript levels were determined to deduce the gene function for *MdCIPK*s in different apple tissues [19,29]. The total RNA was extracted from four different apple organs including roots, leaves, flowers, and fruits. Expression patterns of *MdCIPK* genes were then detected using the qRT-PCR. The analysis results suggested that all the 34 *MdCIPK* genes were expressed in all the tested organs (Figure 5A). From the heat map, compared with other tissues the expression of all *MdCIPK* genes in leaves was significantly lower than in other tissues. The *MdCIPK20* was specifically expressed in roots. *MdCIPK4*, *MdCIPK9*, *MdCIPK15*, and *MdCIPK32* showed high specific expression in flowers, and the *MdCIPK29* showed relatively high expressions in fruit. Notably the *MdCIPK4*, *MdCIPK32*, and *MdCIPK33* showed high expression levels in most tissues except in the leaves. Further to elucidate the tissue specificity of *MdCIPK* genes in apples the *CIPK* genes with high expression levels in each tissue were compared. As shown in the Venn diagram (Figure 5B), *MdCIPK27* and *MdCIPK29* had high expression levels (value > 0.5) in fruit and flower tissue.

*MdCIPK15* had high expression levels in flower (value > 0.5) and leaf (value > 0.05) tissue. Both *MdCIPK10* and *MdCIPK33* had high expression levels (value > 0.5) in fruit, flower, and root tissue. Only the *MdCIPK20* had a high expression level in flower (value > 0.5), fruit (value > 0.5), and leaf (value > 0.05) tissue. In addition, the *MdCIPK4* and *MdCIPK32* had the high expression levels in all four tissues. The strong expression of these *MdCIPK* genes in different tissues implied their important roles in tissue development. This was particularly for the *CIPK4* and *CIPK32* with relative high expression levels in all the tested tissues, which speculated that they might play a key role in the development of apple.

### 2.6. Expression Analysis of MdCIPK Genes at Different Stages in Apple Pollen Tube Growth and Fruit Development

**Figure 6:**
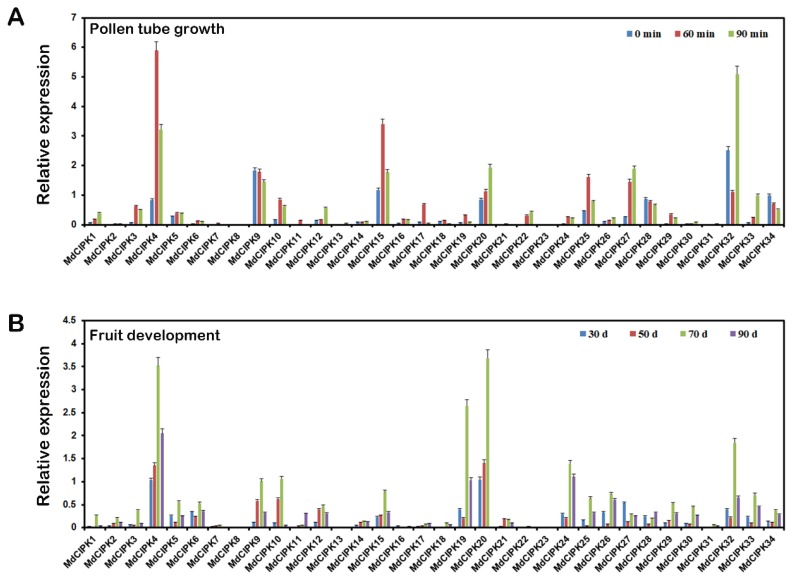
The image summarizes the expression profiles of the 34 *MdCIPK* genes at four stages of pollen tube growth and fruit development. The apple pollen tube at 0, 60 and 90 h after pollination (**A**). The apple fruit harvested at 30, 50, 70 and 90 days after bloom (**B**). Three replicates (1–3) were performed for each stage.

The transcriptome data derived from Illumina RNA-seq reads were used to identify the expression profiles of *MdCIPK* genes during pollen tube growth and fruit development in apple [30,31]. The transcript levels of 34 *MdCIPK* genes were evaluated during three different stages of pollen tube growth. Most of *MdCIPK* genes reveal high variability in transcript levels during apple pollen tube growth (Figure 6A). The *MdCIPK4*, *-9*, *-15*, *-20*, and *-32* were highly expressed at all three pollen tube growth stages, which indicated that these *MdCIPK* genes have regulatory functions throughout pollen tube growth. In contrast, the transcript level of the remaining 29 *MdCIPK* genes were relatively low during these stages. However, some *MdCIPK* genes such as *MdCIPK9*, *-28* and *-34* exhibited decreased expression during the pollen tube growth, which indicated that these *MdCIPK* genes might negatively regulate pollen tube growth. It was worth noting that the expression level of the *MdCIPK4*, *-15*, *-25*, and *-27* showed a significant increase in the early pollen tube growth stage. In particular, the *MdCIPK4* showed specific high expression at the 60 min. In addition, the *MdCIPK34* (the specific high expression was observed at the 90 min) may have a close relationship with the pollen tube growth and flower development.

To identify the expression profiles of *MdCIPK* genes during fruit development in apple, the transcript levels of all 34 *MdCIPK* genes were analyzed by using the Illumina RNA-seq approach. The mRNAs were isolated at 30, 50, 70, and 90 days from developmental apple fruit, and 34 *MdCIPK* genes transcription pattern were evaluated at each stage (Figure 6B). The *MdCIPK4* has the most highly expression in all development stages, which suggested that it might function as a regulator in the fruit development. However, most of the 34 *MdCIPK* genes showed a low transcript level during the four stages. Notably, when the fruit development to 70 days, almost all *MdCIPK* genes transcription levels reached their highest values. Then the gene transcription levels decrease over fruit development time indicating that these *MdCIPK* genes were closely related to fruit development.

### 2.7. Expression Analysis of MdCIPK Genes in Response to Biotic Stress and Abiotic Stress

**Figure 7:**
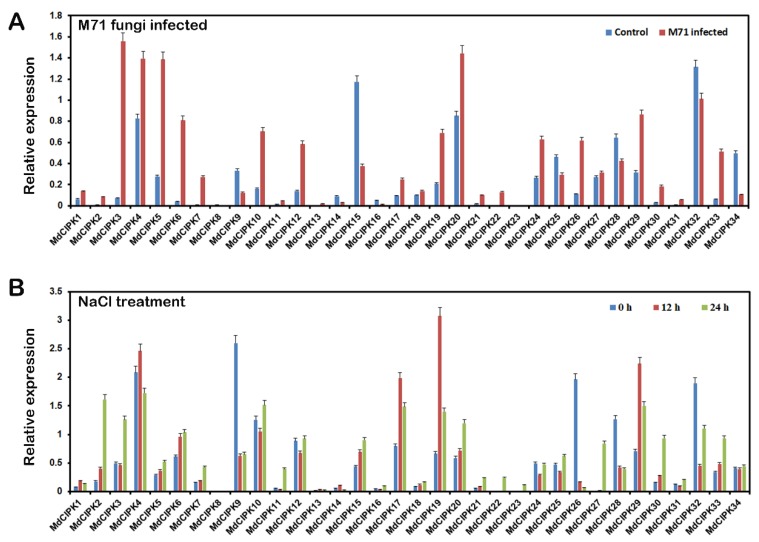
Expression profiles of *MdCIPK* genes under fungal and salt stresses. Shoots with young leaves for fungal treatment and the water used as a control (**A**) or exposed to MS solution containing 100 mM NaCl for salt treatment (**B**) and the samples used for RT-qPCR analysis.

It has been reported that *CIPK* genes were involved in plant response under various stresses [32,33,34]. Therefore, the expression profiles of *MdCIPK* genes under stress condition were analyzed. Transcript levels of all 34 *MdCIPK* genes were characterized. Leaves from apples that were subjected to various stress treatments including biotic stress such as fungal, and abiotic stress such as salt stress (NaCl; 100 mM). All the 34 *MdCIPK* genes in apple were chosen to investigate the responsive patterns of *MdCIPK* genes to fungal and salt stresses (Figure 7).

For fungal treatments, among the 34 *MdCIPK* genes, 23 genes include *MdCIPK1*, *-2*, *-3*, *-4*, *-5*, *-6*, *-7*, *-10*, *-11*, *-12*, *-17*, *-18*, *-19*, *-20*, *-21*, *-22*, *-24*, *-26*, *-27*, *-29*, *-30*, *-31*, *-33* were up-regulated, and 11 genes include *MdCIPK8*, *-9*, *-13*, *-14*, *-15*, *-16*, *-23*, *-25*, *-28*, *-32*, *-34* were down-regulated (Figure 7A). The results showed that all the 34 genes were differentially expressed, and that most of them produced up-regulated responses to biological fungal stress. For salt treatments; among 34 genes, 25 genes include *MdCIPK1, -2*, *-3*, *-4*, *-5*, *-6*, *-7*, *-10*, *-11*, *-12*, *-15*, *-16*, *-17*, *-18*, *-19*, *-20*, *-21*, *-22*, *-23*, *-25*, *-27*, *-29*, *-30*, *-31*, and *-33* were up-regulated, and the remaining 9 genes include *MdCIPK8*, *-9*, *-13*, *-14*, *-24*, *-26*, *-28*, *-32*, and *-34* were down-regulated under salt stress (Figure 7B). Notably most *MdCIPK* genes that simultaneously responded to the two stresses showed diverse divergences expression under fungal or salt stresses, which indicates that the genes may have specific roles in response to these stresses.

## 3. Discussion

*The CIPK* gene family has been reported in Arabidopsis, maize, and rice as contributing to plant development, and has never been reported in apple. In this study, *MdCIPK* genes were identified comprehensively, and the evolutionary relationships, gene structures, chromosomal distribution, and tissue-specific expressions were described and analyzed. In addition, the expression profiles during apple pollen tube growth and fruit development as well as expression profiles have been determined under different stresses treatments.

BLASTP was performed to identify and analyze all *CIPK* genes in the apple genome. 34 *MdCIPK* genes were identified after manual checking. Conserved domain analysis suggested that kinase, NAF, and PPI domains were identified in 34 *MdCIPK* genes (Figure 1), which were *CIPK* family basic characteristics similar to the CIPK proteins in Populus and Arabidopsis [21]. The genome-wide identification results suggested that the number of *CIPK* genes detected in apple was larger than in pear, Arabidopsis, and poplar that can be added to the existing information for *CIPK* genes in apple. Phylogenetic analysis investigated the evolutionary relationships of *CIPK* genes in apple. 34 *MdCIPK* genes were separated into two groups (Group 1 and Group 2), and their diversification during evolution was revealed (Figure 2). The structural analysis suggested that most *MdCIPK* genes contain only one exons with another a small fraction containing multiple exons. Similar gene length and exon/intron were reflected structurers in the same subfamilies. Furthermore, the different lengths of *MdCIPK* might play important roles in the diversification gene functions. (Figure 3). To investigate the *MdCIPK* genomic distribution in apple the locations of *MdCIPKs* were marked on the chromosomes. The chromosomal location analysis revealed that the 34 *MdCIPK* genes were mapped onto a total of 17 apple chromosomes. However, the distribution of *MdCIPK* genes in chromosomes where uneven, indicating that may be related to species evolution and genetic variation (Figure 4).

The *CIPK* genes tissue-specific expression of apple have not been studied previously. In this study, the *MdCIPK* genes expressions in different tissues where investigated (Figure 5). All *MdCIPK* genes displayed different expression patterns, which indicated the distinct effect of *MdCIPK* genes [29]. The *MdCIPK* genes expression patterns were highest in root, flower, and fruit. Higher levels where found in flower and fruit indicating their special roles in pollen tube growth and fruit development [31,35]. The *MdCIPK4* and *MdCIPK32* were strongly expressed in all four tissues, suggesting their potential functions for apple genetic transformation. The different expression patterns shown indicate that they might have various functions in apple development (Figure 6).

Plants have developed unique strategies to adapt to adverse environments [36,37,38,39]. Physiological studies shown that sugars, sugar alcohols, AAs, and amines accumulate in different species in response to various stresses [40]. Recent studies have shown that the protein kinase; *MdCIPK22* interacted with *MdAREB2* in apple, and the *MdCIPK22* are required for ABA-induced phosphorylation at Thr^411^ of the MdAREB2 protein, and enhanced its stability and transcriptional activity [41]. The gene expression of *MdCIPKs* under fungal and salt stresses were explored in this paper with results showing that the *MdCIPK* gene expressions were induced under different treatments (Figure 7). Most of the *MdCIPK* genes were involved in these responses, and where significantly up-regulated indicating that *MdCIPK* genes might participate in the biotic and abiotic stress response [13,22].

In summary, we conducted a genome-wide survey of the *CIPK* family in apple. A total 34 *CIPK* genes were identified in silico analysis base on the apple genome database. They were divided into two groups by the phylogenetic comparison of *CIPK* genes from apple and Arabidopsis. Gene chromosome location demonstrated the evolution of *MdCIPK* genes, and that they contained the common features of exon-intron that are further supported by gene structure analyses. The different expression patterns among different tissues include leaf, root, flower, and fruit revealed their diversified spatiotemporal expression patterns. Most *MdCIPK* genes were expressed in the root, flower, and fruit. Moreover, the expressions of *MdCIPK* genes were analyzed in different pollen tube growth, and fruit development stages further indicating their versatile roles for floral development. Finally, our transcript analysis of *MdCIPK* genes at different biotic and abiotic stress conditions suggest that *MdCIPK* genes are involved in the response of many stresses. Our genome-wide identification and expression analysis provide theoretical reference foundation for functional research of *CIPK* family in apple.

## 4. Materials and Methods

### 4.1. Database Searches and Identification of the CIPK Family in Apple

The database (TAIR, http://www.Arabidopsis.org/) was used to retrieve all the Arabidopsis CIPK protein sequences, and the website GDR (Genome Database for Rosaceae, https://www.rosaceae.org/) was used to search for the apple genome and the version is *Malus x domestica* v3.0.a1 contigs [16]. The BLASTP search (http://blast.ncbi.nlm.nih.gov) was performed using the coding sequences (CDS) and downloaded predicted *CIPK* gene sequences from the GDR database. All the protein sequences were examined from the selected *MdCIPK* candidate genes using the Simple Modular Architecture Research Tool (SMART; http://smart.embl-heidelberg.de/), and domain analysis programs Pfam (http://xfam.org/). In addition, all the redundant and missing sequences of CIPK that without specific domains were removed.

### 4.2. Phylogenetic Analysis and Chromosomal Locations of the CIPK Family

The TAIR website (http://www.Arabidopsis.org/) was used to download the protein sequences. These protein sequences were used as references to categorize the MdCIPK proteins. The website ProtParam (https://web.expasy.org/protparam/) was used to predict protein isoelectric point and molecular weight. MEGA 6.0 (http://www.megasoftware.net/) software with MUSCLE algorithm was used to compare each gene sequences [42]. The phylogenetic analysis was performed using the MEGA 6.0 with neighbor-joining method. The website GDR (Genome Database for Rosaceae, https://www.rosaceae.org/) was used to locate and assign chromosomes and the physical distribution of *MdCIPK* genes was drawn on the apple chromosomes using the MapChart [16].

### 4.3. Plant Materials, Growth Conditions and Treatments

The roots, leaves, stems, flowers, and fruits were collected from 10 years old Gala apple trees (*M. domestica* Borkh.) planted in Northwest A&F University. In May, the flowers of the big balloon period, roots, stems, and leaf tissues were collected, the mature fruits were collected in August, which were used to investigate the tissue-specific expression of *MdCIPK* genes. In vitro shoot cultures were maintained in the culture bottles containing the 3:1 volumetric ratio of soil mix and sand under long-day conditions (14 h light/10 h dark cycle) with a day/night temperature of 25/15 °C in a greenhouse.

To investigate the expression of *MdCIPK* gene in apple fruits at different stages the fruits of apple from pollination to fruit ripening were harvested at 4 different stages of 30, 50, 70, and 90 days respectively. Whole fruits were taken at each developmental stage, then frozen in liquid nitrogen and stored at −80 °C until use. Each biological sample was collected from three different tree and three biological replicates were set at each stage.

To study the expression of *MdCIPK* gene during the apple pollen tube growth, pollen was harvested at 3 different stages at 0, 60, and 90 days from apple pollen tube germination. The pollen tubes were taken at each developmental stage and stored at −80 °C for further analysis. Each biological sample was collected from three different tree and three biological replicates were set at each stage. The CTAB method was used for extracting RNA from triplicate biological replicates samples [43].

To study the effects of biological stress on the expression of *MdCIPK* gene in apple leaves, four weeks old apple leaves (the apple tissue culture seedlings) were treated with the *Alternaria alternaria* f.sp mali M71 fungal fermentation broth [44,45]; sterile water used as the control to treated leaves. The expression analysis of *MdCIPK* gene was investigated when samples treated for 12 h and each biological sample was collected from three different tree and three biological replicates were set at each treatment.

To study the effect of non-biological stress on the expression of *MdCIPK* gene in apple, the apple tissue culture seedlings rooted for half a year which three single-tree per genotype were sprayed with 200 mL, 100 mM NaCl solution or sterile water (control) to drip for the salt stress treatment. During treatments initiation the samples from apple trees were collected on the 0 h, 12 h, and 24 h treatments and were arranged in a completely randomized design with five replicates for each treatment.

### 4.4. RNA Isolation and Quantitative Real-Time PCR

Total RNA was isolated from different tissues of the apple using the CTAB method. The iScript cDNA Synthesis Kit (Bio-Rad, Hercules, CA, USA) was used to reverse-transcribed cDNA from total RNA. Primer premier 5.0 software was used to design all the specific quantitative primers for the *MdCIPK* genes in apple were listed in the supplemental materials (Table A1). Following the manufacturer’s instruction, qRT-PCR was performed on an Icycler iQ5 system (Bio-Rad) using the SYBR Green Supermix Kit (Bio-Rad) and the actin gene was used as controls (Table A1). Compared to untreated plants the relative expression level of each *MdCIPK* gene was calculated as 2^−ΔCT^ values, and each reaction was replicated three times [46].

## Figures and Tables

**Table 1:** The characteristics of *CIPK* family members in apple.

Gene Name	Gene ID	Chromosome	Amino Acids	Intron Number	Isoelectric Point	Molecular Weight (Da)
*MdCIPK1*	MDP0000060056	chr8:25,070,919..25,076,787	448	13	6.91	50,892.45
*MdCIPK2*	MDP0000126770	chr2:23,966,243..23,967,541	433	0	9.61	46,836.24
*MdCIPK3*	MDP0000127732	chr3:5,028,677..5,030,050	462	0	8.78	51,004.81
*MdCIPK4*	MDP0000129860	chr2:1,523,050..1,524,779	527	2	9.68	59,044.53
*MdCIPK5*	MDP0000136806	chr2:3,009,347..3,013,324	515	13	7.68	57,246.77
*MdCIPK6*	MDP0000154855	chr12:25,770,656..25,771,963	436	0	6.81	48,997.12
*MdCIPK7*	MDP0000158876	chr11:4,897,105..4,898,478	458	0	8.64	49,937.29
*MdCIPK8*	MDP0000165729	chr1:21,759,311..21,760,77	497	0	9.52	48,611.3
*MdCIPK9*	MDP0000167597	chr5:6,994,115..6,998,675	529	16	9.21	59,399.19
*MdCIPK10*	MDP0000197160	chr16:16,136,654..16,138,184	441	0	8.97	48,655.04
*MdCIPK11*	MDP0000216765	chr7:18,303,752..18,307,186	475	12	9.04	54,320.72
*MdCIPK12*	MDP0000226074	chr1:26,489,681..26,491,021	447	0	8.66	50,322.2
*MdCIPK13*	MDP0000230405	chr15:10,561,049..10,564,790	322	9	9.27	36,721.29
*MdCIPK14*	MDP0000233288	chr5:7,626,506..7,629,56	458	12	6.92	48,761.51
*MdCIPK15*	MDP0000247429	chr3:19,162,326..19,167,078	433	13	6.14	49,252.67
*MdCIPK16*	MDP0000249604	chr1:22,923,293..22,924,672	460	0	9.16	52,034.2
*MdCIPK17*	MDP0000256347	chr4:17,280,883..17,282,190	436	0	7.98	49,048.48
*MdCIPK18*	MDP0000270573	chr2:3,025,820..3,031,167	564	14	8.37	63,209.44
*MdCIPK19*	MDP0000277672	chr2:1,521,829..1,523,127	433	0	9.05	48,498.09
*MdCIPK20*	MDP0000278839	chr4:17,229,494..17,230,894	467	0	8.08	52,701.59
*MdCIPK21*	MDP0000295392	chr16:20,465,758..20,486,041	604	4	8.34	68,780.45
*MdCIPK22*	MDP0000300274	chr4:18,693,218..18,698,036	538	14	6.56	60,699.97
*MdCIPK23*	MDP0000303485	chr5:5,393,626..5,406,764	729	5	8.37	80,861.01
*MdCIPK24*	MDP0000303735	chr13:11,546,579..11,552,484	471	12	8.71	52,592.3
*MdCIPK25*	MDP0000313460	chr3:5,407,721..5,409,109	463	0	8.88	52,107.05
*MdCIPK26*	MDP0000314872	chr13:13,253,909..13,258,980	437	10	6.17	48,798.65
*MdCIPK27*	MDP0000320872	chr10:27,304,369..27,305,781	471	0	8.80	52,483.4
*MdCIPK28*	MDP0000614281	chr14331:32,383,407..32,384,729	441	0	8.60	49,556.15
*MdCIPK29*	MDP0000632173	chr14975:8,175,823..8,177,121	433	0	9.12	48,593.15
*MdCIPK30*	MDP0000695512	chr5464:5,444,319..5,445,815	499	0	8.21	55,470.78
*MdCIPK31*	MDP0000711750	chr10:27,296,189..27,297,679	497	0	7.58	55,038.17
*MdCIPK32*	MDP0000747045	chr12:25,730,471..25,731,922	484	0	8.71	54,632.11
*MdCIPK33*	MDP0000796828	chr5:5,437,782..5,439,173	464	0	8.82	51,760.44
*MdCIPK34*	MDP0000859587	chr11:5,489,130..5,490,533	468	0	8.85	52,661.9

## Appendix A

Table A1, Information of primers for qPCR.

**Table A1:** Information of primers for qPCR.

Name	Primers (5’-3’)		Group
	Forward Primer	Reverse Primers	
*MdCIPK1*	GATGATGTGAATGCTGTTTT	ATAGTTGCGAATGTGTGTCT	B
*MdCIPK2*	AAGCCCCAGAATCTTTTGC	TTGAGCCGTCGTAGCCG	A
*MdCIPK3*	GAAGGTGGATGTGTGGTC	ACAATCGTCGTCGTAGAAT	A
*MdCIPK4*	CCGTGGTTCTCGTCTGA	TCCTGCTCCTGTTTCGT	A
*MdCIPK5*	ATCCCAATCCCAAAACTCG	TCTGACCGCTCCGCTACA	B
*MdCIPK6*	ATTTTGAAGGACCCGTGG	ACTTGCTAAACAACCCCGA	A
*MdCIPK7*	TCCCAGACAATGCCCT	GCCTTTCACGAACTCCA	A
*MdCIPK8*	TGTCTGGCTTGTTTATGGG	TCTTGTTTCTGCTCACCGT	A
*MdCIPK9*	AGAAGGTCCTCAAGCAC	TCTCCACCAGTCACAAA	B
*MdCIPK10*	CGGCGTCATCCTCTTCG	TCGTGTTTGGGTTCGGG	A
*MdCIPK11*	TTTGTTGATGGGGGTGA	GGAGAAGATTTTCGGGC	B
*MdCIPK12*	GGAACGACGGGCTACT	TCCGCACGACCAGAG	A
*MdCIPK13*	TGATTACCCTATCCCAAG	TCAAGAGTATCTCCTGCG	B
*MdCIPK14*	TTTGGATTGAGTGCCCTA	CCCTTTGTTGTTGATTACCT	B
*MdCIPK15*	CCTAACCCTATGACTCGTG	TTGTTCTTCCTTTTTTTCC	B
*MdCIPK16*	GTGAGGAGGATGTGAGGC	TAATGGCAGTTAGGGTTTG	A
*MdCIPK17*	GGAGGCTTCTGGGCTG	CACGATATGCGGGTGG	A
*MdCIPK18*	GACTTAGGCAACCTCAGC	TTTCCCACAAAAATCACA	B
*MdCIPK19*	CCATAGATTTCTGCCAC	TCGTATCCTTTTTTTCC	A
*MdCIPK20*	TGTTCTATTGGCTGGCTAT	GAGTATTTGGATTGGGGT	A
*MdCIPK21*	CTGACAGACAAAGCCG	GACGATGAATAACCGC	B
*MdCIPK22*	TGGTTACCTGCCTTTT	TCTCTTTCCTTTCCGTT	B
*MdCIPK23*	CGAAAGGTTTGAGTGA	TAAGAAGGTATCCGAGT	A
*MdCIPK24*	GCCAAGGGGAACATAAA	AACCTCGGGAGCAACA	B
*MdCIPK25*	TGGCAACCAAAACTAAGA	ATCCCGATGATAAACACC	A
*MdCIPK26*	AGACCGAAGGCAGGA	GCGCTAAGGCCAAAGT	B
*MdCIPK27*	AGGTCACGCCTTCATTT	GCTGTTGCTGCTCTGTTC	A
*MdCIPK28*	GGAGAAGCGAATCACCAT	GTCGGACTTTTCATCAACA	A
*MdCIPK29*	GCGAGCAAGTCCAAGAT	AAAGCACTGAGTCCGAAA	A
*MdCIPK30*	CTCTGCTTCTGGGTCG	ATCTTGGCTTTCGTGG	A
*MdCIPK31*	GCCCTGCTCTGCTTCT	CCTTGGCGACCTTGTT	A
*MdCIPK32*	AGAAAAAGGAAGTGCGG	CCAGATGAAAAGACGGG	A
*MdCIPK33*	GGTTGCCAAAGGGAAG	CGTGGTGTGGAGGAGG	A
*MdCIPK34*	AGATTATGAAGGTCGGG	TCTTAGTTTTGGTTGCC	A
Actin	TGACCGAATGAGCAAGGAAATTACT	TACTCAGCTTTGGCAATCCACATC	A
